# CD56 in the Immune System: More Than a Marker for Cytotoxicity?

**DOI:** 10.3389/fimmu.2017.00892

**Published:** 2017-07-24

**Authors:** Heleen H. Van Acker, Anna Capsomidis, Evelien L. Smits, Viggo F. Van Tendeloo

**Affiliations:** ^1^Laboratory of Experimental Hematology, Tumor Immunology Group (TIGR), Faculty of Medicine and Health Sciences, Vaccine & Infectious Disease Institute (VAXINFECTIO), University of Antwerp, Antwerp, Belgium; ^2^Cancer Section, UCL Great Ormond Street Institute of Child Health, London, United Kingdom; ^3^Center for Cell Therapy and Regenerative Medicine, Antwerp University Hospital, Edegem, Belgium; ^4^Center for Oncological Research (CORE), Faculty of Medicine and Health Sciences, University of Antwerp, Antwerp, Belgium

**Keywords:** CD56, cellular immune response, interleukin-15, neural cell adhesion molecule, natural killer cell, T cell

## Abstract

Over the past years, the phenotypic and functional boundaries distinguishing the main cell subsets of the immune system have become increasingly blurred. In this respect, CD56 (also known as neural cell adhesion molecule) is a very good example. CD56 is the archetypal phenotypic marker of natural killer cells but can actually be expressed by many more immune cells, including alpha beta T cells, gamma delta T cells, dendritic cells, and monocytes. Common to all these CD56-expressing cell types are strong immunostimulatory effector functions, including T helper 1 cytokine production and an efficient cytotoxic capacity. Interestingly, both numerical and functional deficiencies and phenotypic alterations of the CD56^+^ immune cell fraction have been reported in patients with various infectious, autoimmune, or malignant diseases. In this review, we will discuss our current knowledge on the expression and function of CD56 in the hematopoietic system, both in health and disease.

## Introduction

**Figure 1:**
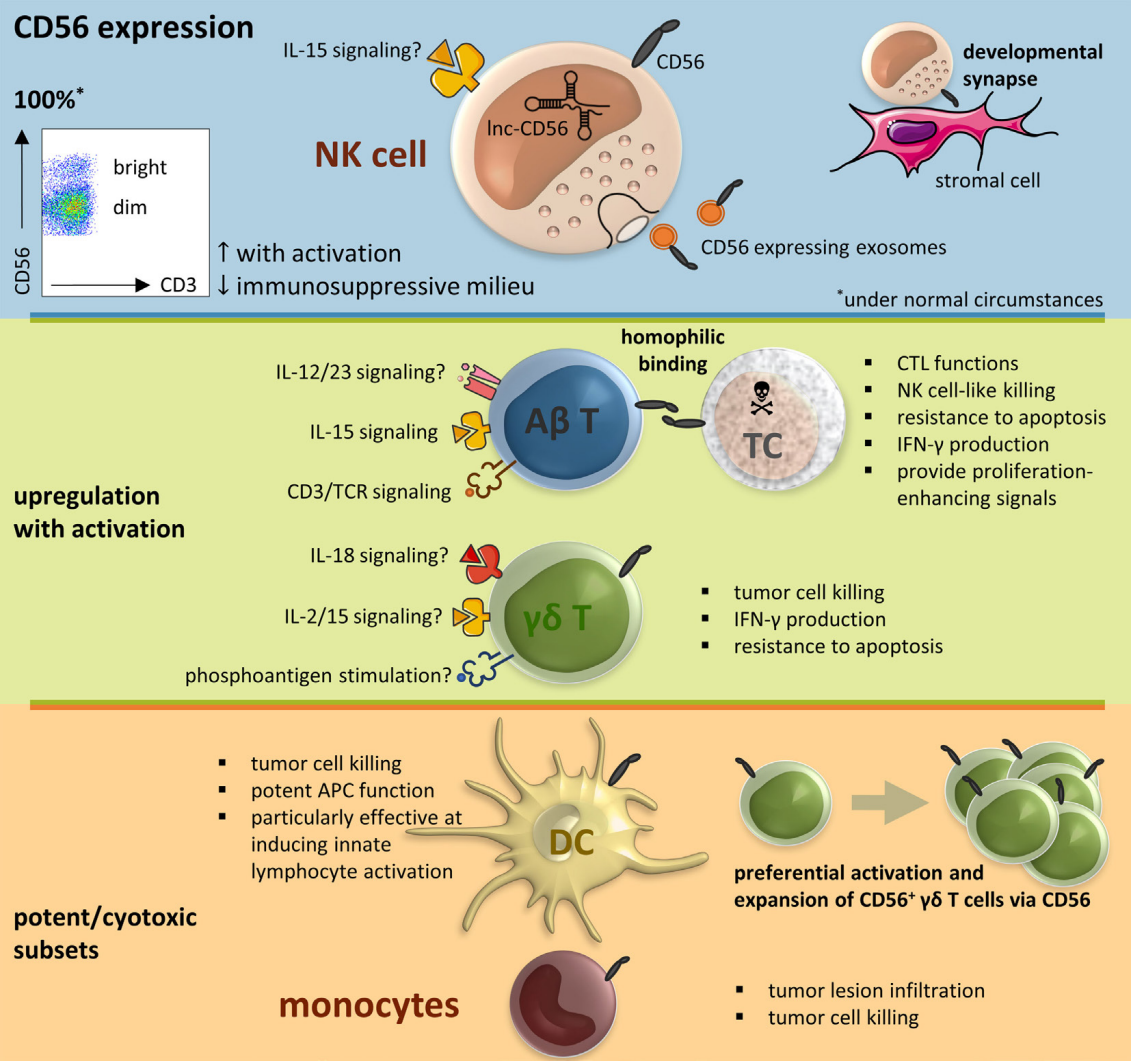
CD56 in the immune system, resumptive figure. Abbreviations: αβ T, alpha beta T cell; γδ T, gamma delta T cell; APC, antigen-presenting cell; CTL, cytotoxic T lymphocyte; DC, dendritic cell; IL, interleukin; IFN, interferon; lnc, long non-coding; NK cell, natural killer cell; SC, stressed cell; TCR, T cell receptor.

The neural cell adhesion molecule (NCAM), also known as CD56, is a member of the immunoglobulin superfamily engaged in both so-called homophilic and heterophilic interactions. Three main isoforms exist of CD56 (NCAM-120, NCAM-140, and NCAM-180), all generated by alternative splicing from one single gene, differing in their intracellular domain length (1). CD56 is often considered a marker of neural lineage commitment due to its discovery site (2). However, CD56 expression is also found in, among others, the hematopoietic system. Here, the expression of CD56 is most stringently associated with, but certainly not limited to, natural killer (NK) cells (Figure 1) (3). CD56 has been detected on other lymphoid cells, including gamma delta (γδ) T cells and activated CD8^+^ T cells, as well as on dendritic cells (DCs) (4–6). Also, in the bone marrow, at the site where hematopoiesis occurs, CD56 fulfills a pivotal role. Mesenchymal stromal cells provide niches for hematopoietic stem cells by, *inter alia*, the expression of adhesion molecules comprising CD56, maintaining long-term hematopoiesis (7, 8). On the other hand, aberrant CD56 expression is seen in a range of hematological malignancies [e.g., multiple myeloma and leukemia (9, 10)] as well as solid tumors [e.g., lung cancer, ovarian cancer, and neuroblastoma (11–13)]. Moreover, numerical and functional deficiencies and phenotypic alterations of CD56^+^ immune cells have been reported in patients with various infectious, autoimmune or malignant diseases (Table 1).

**Table 1:** CD56 expression on immune cells in disease.

Disease	Cell type	Effect	Reference
**Cancer**			

Cancer (general)	Monocytes	↑ higher numbers	(14)
Metastatic melanoma	Alpha beta (αβ) T cells	↑ in patients responding to anti-PD-1 therapy	(15)
(Advanced) solid tumors	αβ T cells	↑ after immunoradiotherapy → injecting CD56^+^ cytotoxic T lymphocytes (CTLs) ↑ complete remissions	(16)
Bladder cancer	Natural killer (NK) cells	↑ in bacillus Calmette–Guérin-treated patients	(17)
Head and neck squamous cell carcinoma	Dendritic cells (DCs)	CD56^+^ DC subsets are absent in metastatic lymph nodes	(18)
Hepatocellular carcinoma	αβ T cells	FOXP3^+^CD3^+^CD4^+^CD56^+^ tumor-infiltrating lymphocytes inversely correlate with survival	(19)
Hepatic metastases of colonic origin	Gamma delta (γδ) T cells	↑ expression of CD56	(20)
Papillary thyroid carcinoma	Monocytes	CD14^+^CD56^+^ monocytes infiltrate into tumor lesions	(14)
Acute myeloid leukemia	γδ T cells	↑ expression of CD56	(6)
Asymptomatic myeloma	NK cells	↑ expression in patients receiving low-dose lenalidomide and DC therapy	(21)

**Infectious diseases**			

HIV	NK cells	Dysfunctional CD56^−^ NK cells	(22)
	αβ T cells	CD56^+^CD8^+^ T cell depletion, but not in natural virus suppressors	(23)
	γδ T cells	CD56^+^ γδ T cell depletion, but not in natural virus suppressors	(24)
Chronic hepatitis C	NK cells	Dysfunctional CD56^−^ NK cells	(25)
	γδ T cells	↑ expression of CD56	(26)
Cytomegalovirus	NK cells	Dysfunctional CD56^−^ NK cells	(27)
Hantavirus	NK cells	Dysfunctional CD56^−^ NK cells	(28)

**Autoimmune disorders**			

Crohn’s disease	Monocytes	↑ higher numbers	(29)
Multiple sclerosis	αβ T cells	Myelin-specific CD56^+^CD4^+^ T cells kill oligodendrocytes	(30)
	αβ T cells	Fingolimod ↑ CD56^+^ T cells in peripheral blood	(31)
Systemic sclerosis	αβ T cells	↑ CD56^+^ CTL recruitment to affected tissues	(32)
Ocular myasthenia gravis	NK cells	Dysfunctional CD56^−^ NK cells	(33)
Psoriasis	αβ T cells	↑ CD56^+^ CTL recruitment to affected tissues	(32)
Rheumatoid arthritis	Monocytes	↑ higher numbers → treatment with etanercept ↓ = better response	(34)

## CD56 Expression on Human Immune Cells in Health and Disease

### NK Cells

Natural killer cells are prototypic members of the innate lymphoid cell (ILC) family and characterized in humans by expression of the phenotypic marker CD56 in the absence of CD3. They are usually further divided into two subsets based on their surface level expression of CD56 (3). Whereas most NK cells in peripheral blood are CD56^dim^, CD56^bright^ NK cells are more abundant in tissues. Until recently it was widely believed that CD56^bright^ NK cells were superior at producing pro-inflammatory cytokines, and CD56^dim^ NK cells were described as the more cytotoxic subset. Rather, CD56^bright^ NK cells respond better to soluble factors, while the CD56^dim^ subset responds better to receptors binding ligands anchored on other cells (35). NK cells have a central role in the cellular immune response, comprising tumor-cell surveillance as demonstrated in the setting of hematopoietic stem cell transplantation (HSCT). The discovery of donor-derived alloreactive NK cells present in T-cell-depleted HLA haploidentical grafts for HSCT was a milestone in the field of NK cell therapy (36–38).

To date, the reason why NK cells, and immune cells in general, express CD56 remains unanswered. However, there is a clear relationship with the degree of activation. Expression of CD56 can therefore be used as a phenotypic activation marker, similar to the use of CD69 and HLA-DR (39–41). For example, upon DC vaccine-mediated activation, both CD56^bright^ and CD56^dim^ NK cell subsets upregulate their expression of CD56 (39, 41). The same applies for expanded NK cells prepared for adoptive transfer using artificial antigen-presenting cells (40). It should therefore come as no surprise that regulatory DCs, in contrast to their mature immunostimulatory counterparts, lack the ability to upregulate the expression of CD56 on CD56^dim^ NK cells (42). Moreover, this applies not only for DC-mediated NK cell stimulation. Upon activation, CD56^dim^ NK cells can adopt a CD56^bright^-like immunophenotype or upregulate their CD56 expression in general (17, 21, 43, 44). Conversely, NK cells exposed to an immunosuppressive milieu downregulate their CD56 expression, concomitant with abolition of their cytotoxicity (45). This is, among others, demonstrated for factors present in plasma of chronic lymphocytic leukemia patients (45). In support of the functional role of CD56, exosomes released by NK cells express CD56 as well (46). Since exosomes are cell-derived vesicles, conceivably having a role in the immune response, the expression of CD56 serves by all odds a purpose, just like the enclosed killer proteins (i.e., FasL, perforin) (46). One possible role could be, for example, the adhesion of the NK cell exosomes to target cells (47).

Lastly, it should be noted that there also exists a CD56^−^ NK cell subpopulation, namely CD3^−^CD4^−^CD14^−^CD19^−^CD16^+^NKp46^+^ lymphocytes (25, 48). While CD56^−^ NK cells are rarely found in healthy individuals (25), elevated levels of CD56^−^ NK cells are commonly found in patients with several pathological conditions, including HIV (22), chronic hepatitis C (25), human cytomegalovirus (27) and hantavirus infections (28), autoimmune disorders (33), and following hematopoietic (stem) cell transplantation (49, 50). In all these diverse settings, CD56^−^ NK cells are reported to be dysfunctional or impaired with reference to their cytolytic capacity and cytokine production. Unfortunately, aging *per se* may also have a deleterious effect on the functional capacity of NK cells. A redistribution of NK cell subsets is confirmed in the elderly, whereby the proportion of the dysfunctional CD56^−^ NK cell subset is increased (51). Altogether, these data emphasize the association between CD56 expression and NK effector function.

### αβ T Cells

The cell-mediated adaptive immune response is primarily attributable to conventional T cells. CD56 expression on these αβ T cells is, similar to NK cells, associated with potent effector function in the human intestine, liver, and peripheral blood (52–54). More specifically, CD56 surface expression on T cells correlates well with expression of CD16, NKG2A/D, NKp44/46, CD122, and DNAM-1, a high intracytoplasmic perforin and granzyme B content, and CD8^+^ cytotoxic T lymphocyte (CTL) functions (23, 53–57). Moreover, CD56^+^ T cells are able to exert NK cell-like killing activity in a pro-inflammatory milieu (54, 57). This property is mainly due to killer cell Ig-like receptor (KIR)^+^ cells within the CD56^+^ T cell fraction (57). All the aforementioned suggests a link between CD56 acquisition by T cells with increased T cell receptor (TCR)-mediated and NK-like cytotoxic potential. Since CD56 also correlates with the expression of the anti-apoptotic protein Bcl-2, increased resistance to apoptosis is advocated (54). Second, CD56^+^ T cells share with NK cells the capacity to produce interferon (IFN)-γ upon interleukin (IL)-15 or IL-12 + IL-18 treatment (53). This pro-inflammatory cytokine production is also seen after stimulation with other immune activating signals such as stimulation of CD3 (4), engagement of the cell adhesion molecule CD2 (LFA-1) (52), or the presence of infectious pathogens. For example, CD56^+^ T cells produce IFN-γ in the presence of *Bacillus Calmette–Guérin*, as well as in response to *Salmonella typhimurium*-infected macrophages (53). This pro-inflammatory cytokine response is, on the other hand, barely detectable in their CD56^−^ counterparts (53). Furthermore, regulatory (IL-10) and T helper 2 (IL-4 and IL-5) cytokine production by CD56^+^ T cells is marginal (4, 52). Next, mucosal CD56^+^ T cells exhibit a compromised proliferation potential as compared to their CD56^−^ counterparts, characteristic for their mature state (52). Nevertheless, CD56^+^ T cells provide proliferation-enhancing signals following global activation to other immune cells, mediating-immune responses in a contact-dependent manner (52).

These properties make CD56^+^ T cells attractive potential targets for therapy for infectious and immune-mediated diseases as well as cancer. For example, in metastatic melanoma, a distinct population of T cells with high expression of HLA-DR and CD56 increased by ninefold in patients responding to anti-PD-1 therapy (15). In line with this, immunoradiotherapy augmented the abundance of circulating CD8^+^CD56^+^ cells in advanced cancer patients (16). Interestingly, injecting patients who failed to respond to immunoradiotherapy with CD56^+^ CTLs into their recurrent metastatic lesions resulted in 59% of complete remissions at these sites (16). On this note, we would like to touch on cytokine-induced killers (CIK) cells that fall within this group of NK cell-like T lymphocytes, whereby the CD56^+^ CIK cells represent the cell type with the highest tumor killing abilities (58, 59). Although CIK cells are beyond the scope of this review, we would like to direct the reader to Schmeel et al. (59) and Mesiano et al. (58) for a comprehensive review.

Proceeding to infectious diseases, CD56-expressing CD8^+^ T cells were found to be depleted in HIV^+^ patients (on therapy), but not in natural virus suppressors, i.e., elite patients suppressing HIV replication without antiretroviral therapy (23). This could be of importance bearing in mind that antiretroviral therapy to date, showing effective HIV suppression and reconstitution of CD4 T cells, still fails to restore CD8^+^ T cell lytic effector functions needed to eradicate the viral reservoir. One reason behind this loss of CD56^+^ T cells could be the high expression of the exhaustion marker TIM-3 on CD56^+^ T cells of HIV patients, whereas this is not seen for CD56^+^ T cells of elite patients. Immune exhaustion is therefore a potential mechanism for preferential depletion of CD56^+^CD8^+^ T cells (23).

Regarding autoimmune diseases, patients suffering from systemic sclerosis, mainly those with active/late capillaroscopic patterns or with severe lung impairment, have decreased numbers of circulating CD56^+^ CTLs as compared to healthy individuals (60). Even though different mechanisms may be involved, this decline in CD56^+^ T cells in the peripheral blood results at least in part from the recruitment and/or trafficking of these cells to the affected tissues, where they cause endothelial cell injury and apoptosis (32). The decrease in CD56^+^ T cell counts is furthermore not only a characteristic of scleroderma but also of other autoimmune disease, for example, of psoriasis (32). In addition, in multiple sclerosis, cytotoxic myelin-specific CD56^+^CD4^+^ T cells were described, killing oligodendrocytes in a NKG2C-mediated manner (30). It should therefore be noted that not only CD56^+^ CTLs but also cytotoxic CD56^+^CD4^+^ T cells do exist, lying at the root of the pathogenesis of disease. As a consequence, the impact of (novel) treatment strategies for multiple sclerosis on the CD56^+^ immune cell fraction is of importance. Fingolimod, a sphingosine 1-phosphate receptor modulator, is indicated as single disease-modifying therapy in highly active relapsing remitting multiple sclerosis patients. Notably, it turns out that fingolimod therapy increases the frequency of CD56^+^ T cells in peripheral blood in multiple sclerosis patients, particularly during relapses (31).

In analogy with NK cells, aging induces weakening of the adaptive immune system, generally termed as immunosenescence (61, 62). This is accompanied by an accumulation of cells combining features of both the innate and adaptive arms of the immune system, most likely to compensate for functional defects of conventional NK and CD8^+^ T cells with age. In contrast to exhausted T cells, senescent terminally differentiated CD45RA (CD8^+^) T (TEMRA) cells are effector T cells with complete competence. They develop features of NK cells comprising the upregulation of NK cell receptors, including CD56. TEMRA cells use their acquired NK cell machinery to maintain rapid effector functions throughout life, tackling the increased burden of tumors and infections in the elderly (61, 62).

As a final remark, not every CD56^+^ T cell exhibits by definition immune invigorating capacity. In the tumor bed of patients with hepatocellular carcinoma, a FOXP3^+^CD3^+^CD4^+^CD56^+^ population with immunosuppressive function has been found (similar to regulatory T cells) (19). In comparison, FOXP3^+^ cells were rarely detected in the CD3^+^CD56^+^ population from adjacent non-cancerous tissues and were completely absent from normal liver tissues (19). The prevalence of FOXP3^+^CD4^+^CD56^+^ T cells in tumor-infiltrating lymphocytes (TILs) was moreover found to be inversely correlated with patient survival (19). Besides FOXP3^+^ TILs, other immune cell subsets may as well exert a powerful regulatory/suppressive influence upon the cell-mediated immune response. In human glioblastoma, a significant proportion of TILs were CD3^+^CD4^+^CD56^+^ immunosuppressive T cells (63). Here too, only a minority of CD3^+^ peripheral lymphocytes expressed CD56 (63). Other non-glial intracranial tumors, including meningioma and metastatic non-small cell lung cancer, showed however no accumulation of CD4^+^CD56^+^ in the tumor (63). It is therefore necessary to take into account that the favorable profile of the CD56^+^ T cells is possibly depending on the T cell subset and/or is sensitive to changes by the tumor microenvironment, with variation between cancer types. Concerning the latter, special emphasis should be given to the expression levels of CD56 on the tumor cells, considering glioblastoma is known to express CD56, whereas, for example, non-small cell lung cancer generally does not.

### γδ T Cells

Gamma delta T cells are the prototype of “unconventional” T cells, defined by the expression of a TCR composed of an γ and δ chain. Although they only constitute less than 5% of all T lymphocytes, their role in the immune system should not be underestimated. γδ T cells have a wide range of functional properties including innate killing, tumor tropism, the support of DC, T cell, and NK cell functions, and, as recently demonstrated, also antigen presentation skills (64–67). Moreover, the proportion of CD56^+^ γδ T cells appears to be determined by their level of activation (6, 20).

Different signals are able to induce γδ T cell activation including phosphoantigens, cytokines, activating receptors, and TCR-mediated signals (65, 68–70). As leading example, stimulation of isolated γδ T cells with isopentenyl pyrophosphate (IPP), a mevalonate-derived isoprenoid phosphoantigen, and the cytokines IL-2 or IL-15 leads to a significant upregulation of CD56 concomitant with CD69 and HLA-DR (6). Activation of γδ T cells with IL-15 and isoprenoid pyrophosphates induces furthermore the expression of IL-15Rα, CD96, CD161, and perforin, all markers of cytotoxic cells (71). Stimulation of γδ T cells from HIV^+^ donors with IL-18 and IPP, results not only in γδ T cell proliferation but also in the higher expression of CD56, NKG2D, and CD107a (72). The cytotoxic phenotype of all these CD56^+^ effector γδ T cells puts forward that CD56 may be a marker of true effector γδ T cells. It is indeed expressed on a potently cytotoxic subset of human γδ T cells. CD56-expressing, but not CD56^−^, IPP-expanded γδ T cells kill head and neck squamous cell carcinoma through the perforin-granzyme pathway (73). Yet, CD56 neutralization itself did not affect CD56^+^ γδ T cell-mediated killing of tumor cells (73). In addition to cytotoxicity, CD56^+^ effector γδ T cells rapidly produce large amounts of IFN-γ upon stimulation (71) and have an increased resistance to Fas ligand and chemically induced apoptosis (73). Strikingly, expression of CD56 is strongest in non-proliferating γδ T cells and gradually disappears with the number of cell divisions (71). Taken together, CD56 defines γδ T cells with increased antitumor activity, identifying a robust γδ T cell subset for effective cancer treatment.

In the context of immunotherapy, expanded γδ T cells for adoptive transfer exhibit an enhanced CD56 expression as well (6). However, this observation was only made in healthy donors. Conversely, γδ T cells of acute myeloid leukemia patients exhibit already elevated levels of CD56, and after expansion, even a downregulation of CD56 is observed (6). The increased expression of CD56 by γδ T cells is also seen in patients with solid tumors, for instance, in γδ T cells associated with hepatic metastases of colonic origin (20), and infectious disease, including chronic hepatitis C virus infection (26). Intriguingly, while the proportion of CD56^+^ γδ T cells dramatically drops in patients with HIV disease, and does not return to normal levels even after prolonged antiretroviral therapy, natural viral suppressors have unaffected CD56^+^ γδ T cell levels and function similar to those of healthy controls (24).

### Dendritic Cells

Given their primary function to capture, process, and present antigens to T cells, DCs play a critical role in stimulating adaptive (antigen-specific) immunity. In general, lytic effector functions are not classically attributed to DCs. However, it is has been demonstrated that both plasmacytoid and myeloid DCs can adapt a CD56^+^ phenotype and acquire cytotoxic functions (5). Plasmacytoid DCs activated by the tick-borne encephalitis vaccine Frühsommer meningoencephalitis display high CD56 expression, coinciding with elevated expression of programmed death-ligand 1, granzyme B, TNF-related apoptosis-inducing ligand (TRAIL), and effector functions. Interestingly, neutralizing CD56 did not result in diminished specific lysis of tumor cells (74). Furthermore, CD56^+^ monocyte-derived IL-15 DCs possess a more pronounced lytic effector function toward tumor cells as compared to their CD56^−^ counterparts, accompanied by elevated TRAIL and granzyme B levels, as well as a superior antigen-presenting capacity (75). Similarly, CD56^+^ IFN-α DCs exhibit a TRAIL-mediated cytolytic activity against tumor cells (76). Apart from exerting a direct cytolytic activity, CD56^+^ DCs have potent antigen-presenting capacity as well (74–76) and are particularly effective at inducing innate lymphocyte activation (41, 77, 78). Unfortunately, malignant cells seem to disable these DC subsets (18, 76). CD56^+^ expression on plasmacytoid and myeloid DCs is downregulated subsequent to contact with head and neck squamous cell carcinoma cells *in vitro*, and CD56^+^ DC subsets are absent in metastatic lymph nodes (18, 76).

### Monocytes

One important function of monocytes is their contribution to the renewal of DCs (and some tissue macrophages) predominantly under inflammatory conditions. CD14^+^CD56^+^ monocytes, which could function as potential precursors of CD56^+^ DCs, have been found in human peripheral blood (14). They are able to infiltrate into tumor lesions and have a direct cytolytic activity toward malignant cells upon activation (14). Interestingly, cancer patients, both with solid tumors and hematological malignancies, reveal much higher numbers of this monocyte subset as compared to healthy controls (14). However, a negative correlation exists between the magnitude of tumor spread and the amount of CD56^+^ monocytes (14). This possibly points to the fact that these cells might play a role in immune tolerance or, in analogy with DCs, that CD56^+^ monocytes are being downregulated by the tumor environment (79). Consonant with the increased prevalence of CD56^+^ monocytes in cancer patients, this subset is also found elevated in autoimmune diseases, such as Crohn’s disease and rheumatoid arthritis (29, 34). From a therapeutic point of view, patients with rheumatoid arthritis treated with etanercept, a TNF-α inhibitor, show a decline in the CD56^+^ monocyte subset, associated with a better response to treatment (34). This is in line with the assumption that CD56^+^ monocytes are being part of the activated cellular immune response.

## CD56 Expression in Other Species

Given the fact that many laboratory animals are used as preclinical models for human disease or basic biomedical research, a brief overview of CD56 expression in other species is warranted, especially since patterns of CD56 expression differ markedly from those found in humans. NK cells of rodents, for example, the most commonly used small animals, do not express CD56 at all. Instead, they are generally identified by their expression of DX5/CD49b or NKR-P1C in mice, or NKR-P1A in rats, in the absence of CD3 (80). Of note, recent data indicate a developmental or lineage relationship between mouse ILCs and human blood CD56^bright^ NK cells, while signature genes of mouse NK cells turned out to be enriched in human CD56^dim^ NK cells (81). However, the current debate on NK cell vs ILC types (82, 83) is beyond the scope of this review. Despite exhibiting far greater similarities to human NK cells, as compared to rodent NK cells, NK cells of non-human primates, such as cynomolgus and rhesus macaques, generally lack CD56 expression as well (84, 85). On the other hand, the majority of monocytes of non-human primates do express CD56 (85, 86). Considering CD56 protein expression on immune cells seems to be highly interspecies dependent, it remains to be addressed whether there is a specific functional role for this molecule on (human) immune cells.

## Upregulation, Binding Interactions, and Functionality

As stated above, there is a body of evidence that CD56 expression is often associated with activation or cytotoxicity in immune cells. This brings forward the question as to whether CD56 is merely an indicator/marker of an activated cell state or if it is actively involved in immune effector function. Moreover, in view of future target discovery for immunotherapy, it is interesting to elucidate which molecular processes give rise to the expression of this molecule. In this context it has been shown that the proportion of the subset of T cells expressing surface CD56 was drastically reduced in IL-12/23 axis-deficient patients, suggesting that the presence of IL-12/23 is mandatory for the expansion of CD56^+^ T cells (53). Of course, this finding does not prove that IL-12/23 stimulation leads to the direct upregulation of CD56, especially since these patients did not show alterations in their CD56^dim/bright^ NK cells subsets (53). On the other hand, culturing NK cells with IL-15 leads to an upregulation of CD56 on both CD56^bright^ and CD56^dim^ NK cells, concomitant with enhanced expression of NK cell activating receptors NKG2D, NKp30, and NKp46 (87). The same shifts in phenotype are also observed in allogeneic HSCT patients, whereby the greatest changes were observed in the early posttransplant months, a period in which IL-15 levels peak (87). In addition, there is evidence that CD56 expression on CD56^−^ T cells can be induced by IL-15 stimulation (54). Purified CD8^+^CD56^−^ T cells express *de novo* CD56 after 12 days of culture with IL-15 (54). Likewise, umbilical cord blood T cells acquire CD56 after culture in IL-15 (56). Therefore, with some certainty, it can be stated that the pleiotropic cytokine IL-15, a well-documented regulator of homeostasis and activation of both innate and adaptive immunity, induces the expression of CD56 on immune cells bearing the IL-2/IL-15Rβ unit such as NK cells and T cells (88, 89). However, the exact mechanism remains unclear. Besides, based on the available literature on this subject, it is unlikely that IL-15 is the only factor capable of having a direct effect on the expression on CD56. For example, similar effects on CD56 expression have been described for CD3/TCR-mediated activation of T cells (4).

On a molecular level, long non-coding (lnc) RNAs orchestrate genetic regulatory outputs, participating in cell differentiation and function (90). Recently, lncRNA AB128931 or lnc-CD56 has been discovered in NK cells, positively correlating with CD56 expression (91). Also, lnc-CD56 knockdown reduces CD56 transcription, providing evidence that lnc-CD56 functions as a positive regulator of CD56 (91). Additional data are however required to unequivocally confirm the roles for this lncRNA in CD56 expression by immune cells in general.

Lastly, from a functional viewpoint, unfortunately, to date, very little is known regarding the functional role of CD56 on immune cells. One key function in the development of NK cells is the CD56-driven migratory behavior of NK cells on stromal cells, forming a developmental synapse (92). NK cells acquire motility with progressive maturation, correlated with the expression of CD56 on developing NK cells. Blocking of CD56 therefore perturbs both NK cell motility and maturation (92). CD56^+^ immune cells are also able to form strong immune synapses with each other through CD56 binding. For example, CD56^+^ DCs have been shown to induce the preferential activation and expansion of CD56^+^ γδ T cells *via* CD56 (93). In particular, homophilic interaction between CD56 molecules on CD56^+^ cells can be formed, including immune cells but also, for example, tumor cells. In this way, CD56^+^ CIK cells are able to kill CD56^+^ leukemic cells (94). This implies that knocking down CD56 on effector cells makes them less cytotoxic against CD56^+^ target cells and, conversely, that downregulating CD56 on target cells impedes CD56-mediated lysis (94).

## Conclusion and Future Perspectives

Hematopoietic expression of CD56 seems to be confined to activated immune cells exhibiting some level of cytotoxic properties. It is therefore tempting to speculate that CD56 is not merely a phenotypic marker of NK cells. αβ T cells, γδ T cells, DCs, monocytes, and possibly even more cells of the immune system can upregulate or neo-express CD56 when activated. This implies some concerns regarding current scientific research. For example, it would be recommended to reconsider the use of negative selection kits making use of CD56 as a depletion marker for NK cells. Chances are that besides the removal of NK cells also other valuable (activated) CD56^+^ white blood cell types will be depleted impacting on the applied assay. Furthermore, in current clinical trials cancer patients with CD56^+^ tumors are being treated with lorvotuzumab mertansine, a CD56-targeting antibody-drug (95). Although in preclinical studies lorvotuzumab was shown to be promising in the treatment of CD56-positive tumors, these results were not entirely translatable to the human situation. A major adverse event was the rate of infection and infection-related deaths upon addition of lorvotuzumab to etoposide/carboplatin therapy in patients with small cell lung cancer (95). The combination of agents with known hematologic toxicities is a risk, but potentially the depletion of CD56^+^ immune cells holds an even greater risk. It is evident that more research is warranted on the role of CD56 expression in cells of the immune system. Clarifying the upregulation and functional role of CD56 on immune cells should therefore be considered a priority, considering current and future immune therapeutic options will most likely benefit from it.

## Author Contributions

HVA researched the data, wrote the review, and designed the figure. AC, ES, and VVT reviewed and revised the manuscript for intellectual content.

## Conflict of Interest Statement

The authors declare that the research was conducted in the absence of any commercial or financial relationships that could be construed as a potential conflict of interest. The reviewer, DR, and handling editor declared their shared affiliation, and the handling editor states that the process nevertheless met the standards of a fair and objective review.

## Abbreviations

CIK, cytokine-induced killers; CTL, cytotoxic T lymphocyte; DC, dendritic cell; γδ T cell, gamma delta T cell; HSCT, hematopoietic stem cell transplantation; IFN, interferon; IL, interleukin; ILC, innate lymphoid cell; IPP, isopentenyl pyrophosphate; KIR, killer cell Ig-like receptor; lnc, long non-coding; NCAM, neural cell adhesion molecule; NK cell, natural killer cell; TCR, T cell receptor; TEMRA, terminally differentiated CD45RA (CD8^+^) T cells; TIL, tumor-infiltrating lymphocytes; TRAIL, TNF-related apoptosis-inducing ligand.
